# Postnatal Intrusive Thoughts and Psychotic-Like Experiences: Exploring Associations with Parenting Experiences and Mental Health

**DOI:** 10.1007/s10597-025-01543-z

**Published:** 2025-11-27

**Authors:** Ilana Foreman, Tammy Hunt, Joanne Peterkin, Joanne Hodgekins

**Affiliations:** 1 Norwich Medical School, Faculty of Medicine and Health Sciences, University of East Anglia, Norwich, United Kingdom; 2 Cambridgeshire and Peterborough NHS Foundation Trust, Cambridge, United Kingdom

**Keywords:** Postnatal, Intrusive thoughts, Psychotic-like experiences, Mental health, Parenting

## Abstract

During the perinatal period, many parents experience mental health difficulties of varying severity, which have been associated with adverse outcomes. Examples include perinatal obsessive-compulsive disorder (OCD) which can be thought to exist on a continuum from subclinical symptoms (e.g., intrusive thoughts (ITs)) to clinical diagnosis of OCD. Similarly postpartum psychosis can range from subclinical ‘psychotic like experiences’ (PLEs) to clinical diagnosis. These disorders are distinct conditions, yet some argue an overlap or comorbidity in symptoms, including co-occurrence postnatally, and they are therefore explored in tandem in this study. Limited literature explores these difficulties in community perinatal populations, and less is known about distress, or potential associations with parenting experiences. A cross-sectional, quantitative design was applied. Participants were parents in the postnatal period (12 months after birth); they completed an anonymous, online survey, exploring experiences of ITs, PLEs, parenting (perceived competence and stress) and mental health (depression, anxiety, and stress). Of 349 participants, 96% reported at least one IT, 90.8% reported associated distress and 95% engaged in behaviours to cope. Considering PLEs, 89% experienced at least one PLE, 88.8% reported associated distress and 30.4% could be considered ‘at-risk’ for developing psychosis. Distressing ITs and PLEs were significantly associated with lower perceived competence and satisfaction, increased parenting stress and mental health symptoms, although this relationship was indirectly mediated by depression and anxiety. Males reported more ITs, parenting stress, depression, anxiety, and lower perceived competence than females. More research is needed to better understand ITs and PLEs across and beyond the perinatal period.

## Introduction

The perinatal period is a time of great change for parents, and estimates suggest over 20% of mothers experience perinatal mental health (PMH) difficulties, which can have adverse outcomes for the mother, the baby and surrounding systems, as well as substantial financial impacts (Bauer et al., 2014; Fawcett et al., 2019). Research typically focuses on perinatal depression and anxiety, given these are commonly experienced (Shorey et al., 2018; Viswasam et al., 2019). However, there is a need for research to explore a broader range of PMH experiences (Howard & Khalifeh, 2020). The postnatal period provides a unique setting in which themes of care and responsibility are activated (Abramowitz et al., 2006) and factors such as sleep deprivation, stress and hormonal changes can impact upon mental health (Lu et al., 2022).

### Perinatal Intrusive Thoughts and Psychotic-Like Experiences

Intrusive thoughts (ITs) are unpleasant, unrealistic, and unwanted thoughts (Abramowitz et al., 2006; Fairbrother & Woody, 2008). ITs are thought to fall on a continuum of obsessive-compulsive disorder (OCD) symptomology, with subclinical ITs being similar in context and form to those seen in OCD, but differing in frequency, intensity, distress and perceived control (Berry & Laskey, 2012; Clark & Rhyno, 2005). Approximately 70–100% of new mothers report ITs (Collardeau et al., 2019), compared to 80–90% of the general population (Clark & Rhyno, 2005). The nature of perinatal ITs vary, though can be related to infant safety (Garcia et al., 2023) and can include distressing thoughts of harm to the infant, whether intentionally or accidentally (Fairbrother et al., 2018; Fairbrother & Woody, 2008). ITs are said to peak in the first few weeks following birth (Brok et al., 2017), possibly due to heightened stress and an increased sense of responsibility (Frias et al., (Frías, et al., 2015)). Many mothers are reluctant to disclose ITs due to feelings of shame (Melles & Keller-Dupree, 2023), although there is no evidence the presence of ITs of infant-related harm is linked to increased risk of infant harm (Abramowitz et al., 2003; Fairbrother et al., 2022). Cognitive models of OCD (Salkovskis, 1999), suggest ITs may be maintained, and develop into clinical OCD, if negative appraisals about the meaning are made (Barrett et al., 2016) and can result in greater distress and impairment (Frías et al., 2015).

Psychotic-like experiences (PLEs) are experiences similar to those seen in psychosis (Ising et al., 2012), including hallucinations and delusional thoughts, but at lower levels of intensity, for example, seeing and hearing things others cannot, or thoughts of being followed. The psychosis continuum suggests psychosis exists on a spectrum (Johns & Van Os, 2001) from milder (subclinical) symptoms such as PLES, to clinical psychosis, which requires support from mental health services. Symptoms along this continuum vary in severity, frequency, associated distress, interpretation, preoccupation, and conviction of beliefs (Derosse & Karlsgodt, 2015; Morrison & Baker, 2000; Peters et al., 2004), and in how individuals appraise and respond to experiences (Johns et al., 2014; Van Os et al., 2009). PLEs have been found to be common during the perinatal period, with 93.5% of mothers experiencing at least one PLE postnatally (Holt et al., 2018), higher than compared to 1–17.5.5% in the general population (Nordgaard et al., 2019). To date, studies have focused on presence of PLEs. Little is known about the frequency of these experiences or their associated distress, yet PLEs are associated with an increased risk of developing psychosis or other mental health difficulties (Lindgren et al., 2022). Therefore, it is vital to better understand the presence, frequency, and associated distress in community perinatal samples (Kaymaz et al., 2012).

OCD and psychotic disorders are distinct conditions, and their relationship is widely debated within the literature, with some highlighting a potential overlap or comorbidity in symptoms, or that one condition may precede or exacerbate the other in some cases (Palermo et al., 2020); ultimately the relationship can be considered complex. Frameworks such as the Hierarchical Taxonomy of Psychopathology (HiTOP) highlight symptom overlap and complexity in diagnosis of mental health conditions (Kotov et al., 2017). To date, ITs and PLEs have largely been reported separately, yet these symptoms have been seen to overlap (Pirec & Grabowski, 2017). PLEs have been found present in individuals with OCD (Korkmaz et al., 2023); longitudinal studies further illustrate the interaction between OCD and psychosis at clinical and subclinical levels (Van Dael et al., 2011; de Haan et al., 2013). Perinatal OCD and postpartum psychosis are met with highest levels of stigma (Cooke et al., 2024) and growing evidence suggests symptoms of OCD and psychosis can co-occur in the postnatal period (Rose et al., 2023). With an increased presence of both PLEs and ITs in the perinatal period, further exploration of their potential interaction is needed. While postnatal PLEs and ITs are not necessarily indicative of mental illness, further understanding of the impact of these experiences, and associated distress is needed to help identify when parents might require further support.

Fathers’ experiences of PMH difficulties have been largely neglected in perinatal research, though can adversely impact upon the father, infant bonding and partner relationships (Philpott, 2023). Consequently, there is a wide variation in reported prevalence rates, which range from 6 to 69%; some suggest the true prevalence is underestimated following under-reporting of paternal PMH symptoms (Paulson et al., 2010). Fathers can experience distressing ITs about their infant (Abramowitz et al., 2003, 2006), yet little is known about their experience of PLEs. In response to calls for perinatal research to be more inclusive (Darwin et al., 2021; Kirubarajan et al., 2022) the current study aimed to recruit *all* parents in the postnatal period.

### Parenting Experiences

The transition to parenthood is a challenging and understandably stressful time, and many parents may question their self-perceived competence and ability to perform the parental role successfully (Deater-Deckard, 1998; Wittkowski et al., 2017). Greater parental competence is linked to academic, social and psychological success in children (Jones & Prinz, 2005), and fewer parental mental health concerns or stress (Kwok & Wong, 2000; Troutman et al., 2012). Notably, parents self-perceived competence is distinctly different from actual parental competence. Greater levels of parenting stress have been linked to poorer child outcomes (Anthony et al., 2005; Curenton et al., 2009), poorer parental mental health outcomes (Redpath et al., 2019) and lower levels of perceived competence in their parental role (Razurel et al., 2017). Evidence suggests that the more severe a parents’ mental illness, the greater the symptom impact is upon parenting stress and perceived parental competence (Kristensen et al., 2024). Parenting stress can be predictive of ITs of intentional harm (Fairbrother & Woody, 2008) and psychosis symptoms (Biaggi et al., 2021). Increased stress is understood to exacerbate ITs and PLEs and vice versa, and the link between stress and psychosis is well established (Xenaki et al., 2024). However, there remains a lack of literature exploring the relationship between non-clinical symptoms such as ITs and PLEs to parenting stress and perceived parental competence, particularly in perinatal and community samples. The perinatal period is a key time for infant development, and it is therefore important to identify parents who may be more likely to experience increased parenting stress, lower perceived competence and need further support.

## Current Study

There are gaps in our understanding about perinatal ITs and PLEs, related distress and associations with parenting experiences, such as perceived parental competence and stress. By better understanding these factors, clinicians will be better able to support the wider perinatal workforce in identifying parents who may need additional support, potentially reducing the risk of more severe mental health difficulties. This study will address the following research questions:

1. How frequent and distressing are ITs and PLEs in the 12 months after having a baby?
2. What factors are associated with increased ITs and PLEs in this period?
3. Are these ITs and PLEs associated with parenting experiences and other mental health symptoms?

## Method

### Participants

A community sample of parents were recruited (January-June 2023) via advertisement on social media and UK-based parenting websites, inviting parents to complete an anonymous online survey about experiences of ‘postnatal unwanted thoughts and unusual experiences’. Participants were eligible if they: self-identified as a parent to an infant aged 12 months or younger; were 16 years old or above, were based in the UK and were proficient in English. No upper age limit was applied, and participants did not need to be first-time parents. Social media advertisements reached 56,831 people, and 1,771 clicked/interacted with the advert.

## Design

A cross-sectional, quantitative design was applied. Ethical approval was granted from the University Faculty of Medicine and Health Sciences Research Ethics Committee.

### Measures

#### Demographic and Background Information

Participants reported their age, gender identity, relationship status, number of conceptions and births, and whether they perceived their birth experience to be traumatic. Participants were asked if they had a history of any mental health difficulties, and if they were in receipt of or awaiting mental health treatment.

***Parental Thoughts and Behaviours Checklist (PTBC)*** (Thiséus et al., 2019).

The PTBC is a self-report questionnaire with 33-items exploring postpartum-specific ITs and 13-items exploring related behaviours. Each item is rated as ‘yes/no/past’ (since birth), with associated time, distress, impairment, resistance, and control rated on a 0–4 Likert scale. This produces a total score (0–20) for thought and behaviour subscales, with higher scores indicating greater symptom severity. The measure can be used with all parents and shows good to excellent internal consistency and psychometric properties (Abramowitz et al., 2006, 2007, 2010; Thiséus et al., 2019). In the current study alpha scores indicate excellent internal consistency α = 0.906.

***Prodromal Questionnaire 16-items (PQ-16)*** (Ising et al., 2012).

The PQ is a 16-item self-report scale which is used as a screening tool for PLEs and can indicate possible psychosis risk. It is not a diagnostic tool and should not be used in isolation to assess for psychosis, instead it can be helpful in identifying PLEs. Sixteen items assess for the presence of positive and negative psychosis symptoms (symptom endorsement). Nine of the 16 items explore perceptual abnormalities and/or hallucinations, five items explore unusual thought content, delusional ideas and/or paranoia, and two items explore mood changes. Each of the 16 items are endorsed on a true/false scale and endorsed items are summed to give a score from 0 to 16. Scores ≥ 6 are indicative of possible psychosis risk, and do not necessarily indicate a diagnosis of psychosis is warranted; therefore participants with scores ≥ 6 were categorised as a ‘potential risk’ group, and those with scores < 6 were categorised as a ‘low/no risk’ group. Distress is rated for endorsed items on a 0 (none) to 3 (severe) scale and summed to give a total distress score (range 0–48), with higher scores indicative of greater distress. We acknowledge the PQ-16 is not specific to the perinatal period, and some items may overlap with ‘normal’ perinatal symptoms, however given a scarcity of alternative perinatal psychosis measures available during data collection, it was felt the PQ-16 would be appropriate, it has also been used in perinatal (Levey et al., 2018; Adjorlolo et al., 2022) and non-help seeking (community) populations (Howie et al., 2022). It has good psychometric properties and internal consistency (de Jong et al., 2021; Ising et al., 2012). In the current study alpha scores indicate good internal consistency α = 0.831. To capture postnatal experiences, parents were asked to complete the measure based on the last 12 months since becoming a parent.

***Parenting Sense of Competence Scale (PSOC)*** (Gibaud-Wallston & Wandersman, 1978).

This 17-item self-report scale consists of two subscales: eight items measure perceived parental self-efficacy (self-perceived competence in the parenting role), and nine items measure parental satisfaction (liking of the parental role). Items are rated on a 6-point Likert scale (1: ‘strongly disagree’ to 6: ‘strongly agree’). Nine items are reverse scored. Summed scores create an overall total score (range 26–102), and a score for each subscale, with higher scores indicative of higher perceived parental competence. Good reliability and validity have been found (Gibaud-Wallston & Wandersman, 1978; Gilmore & Cuskelly, 2009; Johnston & Mash, 1989). In the current study alpha scores indicate good internal consistency α = 0.872.

***Parental Stress Scale (PSS)*** (Berry & Jones, 1995).

The PSS is an 18-item self-report measure of parental stress. Participants rate their level of agreement on each item, using a 5-point likert scale (1: ‘strongly disagree’ to 5: ‘strongly agree’). Summed scores provide a total score between 18 and 90, with higher scores indicative of higher levels of parental stress. The PSS has been found to have good reliability and validity (Algarvio et al., 2018; Berry & Jones, 1995). In the current study alpha scores indicate good internal consistency α = 0.896.

***Depression Anxiety Stress Scale 21-item (DASS-21)*** (Lovibond & Lovibond, 1995).

The DASS-21 is a 21-item self-report measure assessing depression, anxiety, and stress symptoms. Participants use a 4-point scale to rate the extent to which they have experienced each item in the past week (0: ‘did not apply to me at all’ to 3: ‘applied to me very much, or most of the time’). Total scores range from 0 to 42, with higher scores indicative of greater symptom severity. Scores within the ‘moderate’ and above range can be considered a severity cut-off for clinical symptoms (depression: 14+; anxiety: 10+; stress: 19+). The DASS-21 is recommended for use in non-clinical and postnatal populations (Meades & Ayers, 2011; Miller et al., 2006; Xavier et al., 2016). It shows convergent and divergent validity (Miller et al., 2006), excellent reliability (Osman et al., 2012), excellent criterion validity, and good to excellent internal consistency (Gloster et al., 2008). In the current study alpha scores indicate excellent internal consistency α = 0.949.

## Procedure

The study advert was shared on UK parenting sites (Mumsnet, Netmums, Dads Matter UK), and social media (Facebook, Twitter, and Instagram) where targeted advertising was applied. The survey was distributed via the Jisc Online Surveys platform, where participants were presented with the information sheet and consent form, prior to completing the study measures. A debrief form provided details of support resources. A prize draw for vouchers was offered as remuneration for participants’ time. Average completion time was 24 min.

### Analysis

Data were analysed using IBM SPSS Statistics Version 28. Statistical analysis was completed using two-tailed analysis and *p* <.05 alpha level. Data were screened for parametric requirements and assumptions tested, with no violations identified. Descriptive statistics include frequencies and percentages for categorical variables and mean and standard deviation for continuous variables. Mean comparison, correlational, multiple regression and mediation analyses were run to answer the research questions.

## Results

### Demographic Information and Descriptive Statistics

A total of 349 parents completed the survey. There were 475 births (*M* = 1.36), and 670 conceptions (*M* = 2) reported, indicating 36.2% of the sample had experienced possible pregnancy loss. 20.3% participants (*N* = 72) had both a history of mental health difficulties and were awaiting/receiving mental health treatment. Table 1 details demographic and background information and Table 2 details descriptive statistics for study variables.

**Table 1:** Participant demographic and background information

	Frequency (%)		
	All Parents	Potential Risk*	No/Low Risk**
N	349	106	243
Age			
16–19	0 (0)	0 (0)	0 (0)
20–24	33 (9.5)	15 (14.2)	18 (7.4)
25–29	127 (36.4)	53 (50.0)	74 (30.5)
30–34	134 (38.4)	29 (27.4)	105 (43.2)
35–39	44 (12.6)	7 (6.6)	37 (15.2)
40–44	11 (3.2)	2 (1.9)	9 (3.7)
45+	0 (0)	0 (0)	0 (0)
Gender			
Female	316 (90.5)	89 (84)	227 (93.4)
Male	28 (8)	14 (13.2)	14 (5.8)
Non-Binary	3 (0.9)	2 (1.9)	1 (0.4)
Transgender	1 (0.3)	0 (0)	1 (0.4)
Prefer not to say	1 (0.3)	1 (0.9)	0 (0)
Relationship Status			
Single	13 (3.7)	6 (5.7)	7 (2.9)
In a relationship, not cohabiting	18 (5.2)	8 (7.5)	10 (4.1)
Cohabiting	144 (41.3)	51 (48.1)	93 (38.3)
Married	171 (49.0)	38 (35.8)	133 (54.7)
Divorced/Separated	1 (0.3)	1 (0.9)	0 (0)
Civil Partnership	2 (0.6)	2 (1.9)	0 (0)
History of mental health difficulties ^a^	169 (48.4)	66 (62.3)	101 (42.4)
Currently receiving or awaiting treatment for mental health difficulty ^a^	88 (25.2)	41 (38.7)	47 (19.3)
Perception of birth as traumatic ^a^	174 (49.9)	62 (58.5)	112 (46.1)
Previous pregnancy loss ^a, b^	119 (36.1)	46 (39.3)	73 (34.3)
	**Mean (SD)**		
Number of births	1.36 (0.68)	1.37 (0.63)	1.36 (0.7)
Number of conceptions	2.01 (1.556)	2.09 (1.68)	1.95 (1.47)

^a^ Reflects the number and percentage of participants answering “yes” to this question

^b^ Previous pregnancy loss indicated by a higher number of conceptions than births

*Potential risk refers to participants scoring 6 or above on the PQ-16.

** No/low risk refers to participants scoring below 6 on the on PQ-16.

**Table 2:** Descriptive statistics of study variables

	All Parents (*N* = 349)	Potential Risk* (*N* = 106)		Low/No Risk** (*N* = 243)	
	**Mean (SD)**				
PQ-16					
PLE Symptom Endorsement	4.34 (3.53)	8.78 (2.50)		2.40 (1.67)	
PLE Distress Score	6.37 (7.09)	14.27 (7.45)		2.92 (2.99)	
PTBC					
PTBC Thoughts Score	6.42 (3.36)	8.62 (3.17)		5.47 (2.97)	
PTBC Behaviours Score	5.96 (3.61)	7.92 (3.61)		5.11 (3.26)	
DASS					
Depression Score	13.42 (10.91)	20.70 (10.38)		10.25 (9.53)	
Anxiety Score	10.46 (9.54)	16.85 (9.87)		7.68 (7.93)	
Stress Score	19.51 (10.0)	26.08 (8.91)		16.63 (9.18)	
PSOC					
Total	65.95 (13.44)	59.39 (12.44)		68.81 (12.86)	
Satisfaction	33.06 (8.02)	28.94 (7.57)		34.86 (7.55)	
Self-Efficacy	32.88 (8.08)	30.45 (7.86)		33.94 (7.97)	
PSS					
PSS Total	45.60 (11.33)	50.01 (11.64)		43.67 (10.65)	
	**Frequency (%)**				
PLEs					
Reported at least one PLE	313 (89.7)		106 (100)		198 (81.5)
Reported experiencing distress	310 (88.8)		106 (100)		185 (76.1)
ITs					
Reported at least one IT	336 (96.3)		105 (99.1)		231 (95.1)
Experienced distress from ITs	317 (90.8)		104 (98.1)		213 (87.6)
Depression Score					
Below clinical cut-off	154 (44.1)		14 (13.2)		140 (57.6)
Above depression cut-off	195 (55.9)		92 (86.908)		103 (42.4)
Mild	33 (9.5)		11 (10.4)		22 (9.1)
Moderate	78 (22.3)		33 (31.1)		45 (18.5)
Severe	40 (11.5)		23 (21.7)		17 (7.0)
Extremely Severe	44 (12.6)		25 (23.6)		19 (7.8)
Anxiety Score					
Below clinical cut-off	164 (47)		19 (17.9)		145 (59.7)
Above anxiety cut-off	185 (53)		87 (82.1)		98 (40.3)
Mild	19 (5.4)		4 (3.8)		15 (6.2)
Moderate	68 (19.5)		27 (25.5)		41 (16.9)
Severe	32 (9.2)		12 (11.4)		20 (8.2)
Extremely Severe	66 (18.9)		44 (41.5)		22 (9.1)
Stress Score					
Below clinical cut-off	136 (39)		10 (9.4)		126 (51.9)
Above stress cut-off	213 (61)		96 (90.6)		117 (48.1)
Mild	39 (11.2)		12 (11.3)		27 (11.1)
Moderate	63 (18.1)		26 (24.5)		37 (15.2)
Severe	76 (21.8)		34 (32.1)		42 (17.3)
Extremely Severe	35 (10)		24 (22.6)		11 (4.5)

Note. *PQ-16* Prodromal Questionnaire-16-items (symptom endorsement and distress), *PTBC* Parental Thoughts and Behaviours Checklist (thoughts and behaviour scales), *DASS-21* Depression Anxiety Stress Scale (Depression, anxiety and stress clinical cut-off’s refer to those specified in the DASS-21), *PSOC * Parental Sense of Competency Scale (Self-Efficacy and Satisfaction Subscales), *PSS* Parental Stress Scale

*Potential risk refers to participants scoring 6 or above on the PQ-16

** No/low risk refers to participants scoring below 6 on the on PQ-16

### Frequency and Distress

Tables 3, 4 and 5 detail frequencies of PTBC and PQ-16 items.

**Table 3:** PTBC frequencies

Thoughts Items	Yes %	Past %	Total *N* (%)
1. Stop breathing	79.9	13.5	326 (93.4)
2. Getting smothered	40.7	14.0	191 (54.7)
3. Suffocate while sleeping	70.5	14.9	298 (85.4)
4. Sudden infant death syndrome	72.5	16.9	312 (89.4)
5. Burp too hard	32.4	17.2	173 (49.6)
6. Scream, shake, or slap	30.7	14.9	159 (45.6)
7. Purposely drown	6.3	2.3	30 (8.6)
8. Stabbing baby	6.3	2.9	32 (9.2)
9. Burning with hot water	10.3	4.3	51 (14.6)
10. Mistakenly puncturing soft spot	28.1	14.3	148 (42.4)
11. Accidental death	65.9	11.5	270 (77.4)
12. Dropping baby	69.9	12.3	287 (82.2)
13. Dropping from height	30.9	8.0	136 (39)
14. Injured if picked up wrong	50.7	15.2	230 (65.9)
15. Choking	72.8	7.4	280 (80.2)
16. Animal attack	49.9	10.9	212 (60.7)
17. Drowning during a bath	35.2	12.9	168 (48.1)
18. Car accident involving the baby	64.8	8.6	256 (73.4)
19. Parent hurt/absent	74.8	8.6	291 (83.4)
20. Forget baby in car seat	23.8	4.6	99 (28.4)
21. Give the baby away	16.6	6.0	79 (22.6)
22. Baby taken away	49.0	7.2	196 (56.2)
23. Leaving the baby when crying	32.1	7.4	138 (39.5)
24. Sick from the floor/unclean surfaces	34.1	5.7	139 (39.8)
25. Sick from bodily waste	18.9	4.6	82 (23.5)
26. Concerns about household items	35.8	5.4	144 (41.3)
27. Concerns about animals or insects	28.4	5.2	117 (33.5)
28. Concerns about contamination	34.1	6.6	142 (40.7)
29. Thoughts about genitals	8.0	4.0	42 (12)
30. Thoughts about sexual orientation	20.9	4.0	87 (24.9)
31. Sexual breastfeeding thoughts	7.2	2.9	35 (10)
32. Other sexual thoughts	6.6	1.7	29 (8.3)
33. Medical illness/disease fears	39.0	7.7	163 (46.7)
Behaviours Items	**Yes %**	**Past %**	**Total N (%)**
1. Reassurance	90.3	5.2	333 (95.4)
2. Rationalise	73.1	6.0	276 (79.1)
3. Checking	86.0	7.4	326 (93.4)
4. Distraction activities	65.6	4.0	258 (73.9)
5. Distraction thoughts	75.9	4.0	279 (79.9)
6. Thought suppression	77.1	3.4	281 (80.5)
7. Avoid situations	43.6	7.7	179 (51.3)
8. Avoid baby	9.5	5.4	52 (14.9)
9. Get social support	52.7	8.9	215 (61.6)
10. Ask others if normal	37.2	8.9	161 (46.1)
11. Confess thoughts	38.7	9.2	167 (47.9)
12. Pray	15.2	1.1	57 (16.3)
13. Other strategies	33.0	2.9	125 (35.8)

**Table 4:** PTBC ‘Thoughts’ and ‘Behaviours’ Follow-Up questions

Thoughts Items	Response	*N* (%)
1. Time occupied by thoughts	None	20 (5.7)
	Less than 1 h per day/occasional thoughts	197 (56.4)
	1–3 h per day/frequent thoughts	100 (28.7)
	3–8 h per day/very frequent thoughts	29 (8.3)
	More than 8 h per day/near constant thoughts	3 (0.9)
2. Interference of thoughts	None	130 (37.2)
	Slight interference but overall performance not impaired	120 (34.4)
	Definite interference, but still manageable	83 (23.8)
	Causes substantial impairment in performance	16 (4.6)
	Incapacitating	0 (0)
3. Distress from thoughts	None	32 (9.2)
	Not too disturbing	85 (24.4)
	Disturbing but still manageable	185 (53)
	Very disturbing	43 (12.3)
	Near constant disabling distress	4 (1.1)
4. Effort to resist thoughts	Always make an effort to resist	143 (41)
	Try to resist most of the time	129 (37)
	Make some effort to resist	57 (16.3)
	Yield to thoughts without attempting to resist, but with reluctance	18 (5.2)
	Completely and willingly yield to all the thoughts	2 (0.6)
5. Control over thoughts	Complete control	47 (13.5)
	Much control, usually able to stop/divert the thoughts	148 (42.4)
	Moderate control, sometimes able to stop or divert thoughts	109 (31.2)
	Little control, rarely successful in stopping or dismissing thoughts	39 (11.2)
	No control, I am unable to even temporarily alter them	6 (1.7)
Behaviour Items	**Response**	**N (%)**
1. Time spent engaging in strategies	None	23 (6.6)
	Less than 1 h per day/occasionally	226 (64.8)
	1–3 h per day/frequently	81 (23.2)
	3–8 h per day/very frequently	16 (4.6)
	More than 8 h per day/near constantly	3 (0.9)
2. Interference from strategies	None	161 (46.1)
	Slight interference but overall performance not impaired	122 (35)
	Definite interference, but still manageable	55 (15.8)
	Causes substantial impairment in performance	11 (3.2)
	Incapacitating	0 (0)
3. Prevented from performing strategies	None	70 (20.1)
	Not too disturbing	99 (28.4)
	Disturbing but still manageable	118 (33.8)
	Very disturbing	48 (13.8)
	Near constant disabling distress	14 (4)
4. Resistance to performing strategies	Always make an effort to resist	127 (36.4)
	Try to resist most of the time	96 (27.5)
	Make some effort to resist	96 (27.5)
	Yield to thoughts without attempting to resist, but with reluctance	21 (6)
	Completely and willingly yield to all the thoughts	9 (2.6)
5. Control over drive to perform strategies	Complete control	83 (23.8)
	Much control, usually able to stop/divert the behaviours	133 (38.1)
	Moderate control, sometimes able to stop or divert behaviours	93 (26.6)
	Little control, rarely successful in stopping or diverting behaviours	34 (9.7)
	No control, drive to perform behaviours is overpowering, rarely able to even delay performance	6 (1.7)

**Table 5:** PQ-16 item endorsement and distress frequencies

Item		Endorsement *N* (%)	Distress Rating *N* (%)	
1. I feel uninterested in the things I used to enjoy		175 (50.1)	None Mild Moderate Severe	19 (5.4) 70 (20.1) 66 (5.7) 20 (5.7)
2. I often seem to live through events exactly as they happened before (Déjà Vu)		125 (35.8)	None Mild Moderate Severe	34 (9.7) 43 (12.3) 35 (3.7) 13 (3.7)
3. I sometimes smell or taste things that other people can’t smell or taste		111 (31.8)	None Mild Moderate Severe	39 (11.2) 48 (13.8) 19 (5.4) 5 (1.4)
4. I often hear unusual sounds like banging, clicking, hissing, clapping, or ringing in my ears		120 (34.4)	None Mild Moderate Severe	23 (6.6) 51 (14.6) 39 (11.2) 6 (1.7)
5. I have been confused at times whether something I experienced was real or imaginary		138 (39.5)	None Mild Moderate Severe	12 (3.4) 55 (15.8) 56 (16.0) 15 (4.3)
6. When I look at a person, or look at myself in a mirror, I have seen the face change right before my eyes		33 (9.5)	None Mild Moderate Severe	4 (1.1) 11 (3.2) 14 (4.0) 4 (1.1)
7. I get extremely anxious when meeting people for the first time		172 (49.3)	None Mild Moderate Severe	1 (0.3) 58 (16.6) 83 (23.8) 30 (8.6)
8. I have seen things that other people apparently can’t see		37 (10.6)	None Mild Moderate Severe	1 (0.3) 12 (3.4) 17 (4.9) 7 (2.0)
9. My thoughts are sometimes so strong that I can almost hear them		109 (31.2)	None Mild Moderate Severe	11 (3.2) 31 (8.9) 46 (13.2) 31 (6.0)
10. I sometimes see special meanings in advertisements, shop windows, or in the way things are arranged around me		51 (14.6)	None Mild Moderate Severe	14 (4.0) 27 (7.7) 9 (2.6) 1 (0.3)
11. Sometimes I have felt that I’m not in control of my own ideas or thoughts		142 (40.7)	None Mild Moderate Severe	7 (2.0) 48 (13.8) 64 (18.3) 23 (6.6)
12. Sometimes I feel suddenly distracted by distant sounds that I am not normally aware of		89 (25.5)	None Mild Moderate Severe	19 (5.4) 46 (13.2) 20 (5.7) 4 (1.1)
13. I have heard things other people can’t hear like voices of people whispering or talking		44 (12.6)	None Mild Moderate Severe	3 (0.9) 19 (5.4) 15 (4.3) 7 (2.0)
14. I often feel that others have it in for me		105 (30.1)	None Mild Moderate Severe	3 (0.9) 36 (10.3) 46 (13.2) 20 (5.7)
15. I have had the sense that some person or force is around me, even though I could not see anyone		64 (18.3)	None Mild Moderate Severe	14 (4.0) 23 (6.6) 21 (6.0) 6 (1.7)
16. I feel that parts of my body have changed in some way, or that parts of my body are working differently than before		179 (51.3)	None Mild Moderate Severe	28 (8.0) 74 (21.2) 59 (16.6) 19 (5.4)
**Frequency**	**Item**			**N (%)**
How frequently do these thoughts, ideas or experiences occur?	None			78 (22.3)
	Less than 1 h per day, or occasionally			159 (45.6)
	1 to 3 h per day, or frequently			84 (24.1)
	3 to 8 h per day, or very frequently			20 (5.7)
	More than 8 h per day, or near constantly			8 (2.3)

### ITs

Over 96% of participants reported at least one current IT and 90.8% experienced related distress. The most frequently endorsed (93.4%) was “thoughts baby might stop breathing”. Of 349 parents, 94% experienced ITs each day; 62.8% felt ITs interfered with daily functioning; 59.1% had to make effort to resist ITs; and 13.5% felt they had complete control over their ITs. Considering IT-related behaviours, 95% of parents engaged in at least one coping behaviour, and 54% felt these interfered with daily functioning, the most frequently reported was ‘reassurance seeking’ (95.4%). 80% indicated they would feel distressed if unable to perform strategies; 63.6% had to make effort to resist performing strategies and 76.1% had a strong drive to perform strategies.

### PLEs

Over 89% of participants reported having experienced at least one item on the PQ-16, 77.7% indicated these occurred at least occasionally each day, and 88.8% reported associated distress. The item “I feel that parts of my body have changed in some way, or that parts of my body are working differently than before”, was most frequently endorsed (51.3%) and excluded from scoring, in terms of the psychosis risk cut-off, as it was likely to be linked to the physical changes occurring after birth. The most frequently endorsed items were two related to mood: “I feel uninterested in the things I used to enjoy” (50.1%), and “I get extremely anxious when meeting people for the first time” (49.3%). When excluding these mood items; lower percentages of participants endorsed at least one item relating to attenuated psychotic symptoms (81.8%): 63.9% endorsed at least one perceptual experience and 71% endorsed at least one paranoid or delusional experience. We recognise other items could be interpreted as being related to ‘normal’ perinatal or other mental health experiences, however, to maintain validity of the measure, no further items were omitted from scoring or interpretation.

#### Potential Risk Group

Over 30% of participants endorsed ≥ 6 items on the PQ-16 and could be considered a ‘potential risk’ group; parents who scored below this cut-off were categorised as being at low/no risk of developing psychosis. A high proportion of the potential risk group scored in the clinical range for depression (86.8%), anxiety (82.1%) and stress (90.6%), (see Tables 1 and 2 for comparisons).

MANOVA analyses compared scores between the potential risk and low/no risk groups for ITs, PLEs, perceived competence, parenting stress and depression, anxiety and stress (Table 6). The differences in scores across all measures were statistically significant (*p* <.001). Therefore, the potential risk group experienced significantly more PLEs, distress from PLEs, ITs and related behaviours, lower perceived competence, increased parenting stress and symptoms of depression, anxiety, and stress than the group of parents not at risk of psychosis.

**Table 6:** MANOVA results for differences between potential risk and low/no risk groups

	Potential Risk* M (SD)	Low/No Risk** M (SD)	F Value	*p*	η_*p*_^2^
PLE	8.78 (2.50)	2.40 (1.67)	778.76	< 0.001	0.692
PLE Distress	14.27 (7.45)	2.92 (2.99)	411.83	< 0.001	0.543
PTBC Thoughts	8.65 (3.17)	5.47 (2.97)	79.96	< 0.001	0.187
PTBC Behaviours	7.92 (3.61)	5.11 (3.26)	51.13	< 0.001	0.128
Competence	59.39 (12.44)	68.81 (12.86)	40.32	< 0.001	0.104
Parenting Stress	50.01 (11.64)	43.67 (10.65)	24.66	< 0.001	0.066
Depression	20.70 (9.53)	10.25 (9.53)	83.95	< 0.001	0.195
Anxiety	16.85 (9.87)	7.68 (7.93)	84.59	< 0.001	0.196
Stress	26.08 (8.93)	16.64 (9.18)	79.21	< 0.001	0.186

Note. *PTBC* Parental Thoughts and Behaviours Checklist (thoughts and behaviour scales)

*Potential risk refers to participants scoring 6 or above on the PQ-16

** No/low risk refers to participants scoring below 6 on the on PQ-16

### Factors Associated with Increased its and PLEs in this Period

Separate one-way MANOVA analyses indicated significant differences in ITs and PLEs, which were more commonly experienced in parents with a history of mental health problems, those awaiting or receiving mental health treatment and those with birth trauma. Table 7 details full results.

**Table 7:** MANOVA results for differences in experiences between parents with mental health history, mental health treatment, birth trauma and by gender

	M (SD)		F Value	*P*	η_*p*_^2^
	Mental Health History				
	Yes	No			
ITs	7.38 (3.31)	5.52 (3.15)	28.92	< 0.001	0.077
PLEs	5.37 (3.79)	3.37 (2.96)	30.31	< 0.001	0.080
	**Mental Health Treatment**				
	Yes	No			
ITs	8.33 (3.26)	5.78 (3.14)	42.33	< 0.001	0.109
PLEs	6.29 (4.32)	3.68 (2.94)	40.06	< 0.001	0.104
	**Birth Trauma**				
	Yes	No			
ITs	6.79 (3.35)	6.06 (3.34)	4.09	0.044	0.012
PLEs	4.79 (3.88)	3.88 (3.53)	5.91	0.016	0.017
	**M (SD)**		**F Value**	**P**	**η**_**p**_^**2**^
	**Gender**				
	Female (*N* = 316)	Male (*N* = 28)			
ITs	6.22 (3.38)	8.11 (2.27)	8.45	0.004*	0.024
IT Behaviours	5.71 (3.58)	8.18 (3.12)	12.45	< 0.001*	0.035
PLE Endorsement	4.21 (3.45)	5.64 (4.08)	4.33	0.038	0.012
PLE Distress	6.12 (6.98)	8.64 (7.25)	3.37	0.067	0.010
Parental Competence	66.92 (13.27)	55.89 (10.74)	18.29	< 0.001*	0.051
Parenting Stress	44.47 (10.87)	56.93 (10.06)	34.19	< 0.001*	0.091
Depression	12.64 (10.64)	20.93 (10.35)	15.67	< 0.001*	0.044
Anxiety	9.90 (9.10)	16.21 (12.20)	11.65	< 0.001*	0.033
Stress	19.16 (10.06)	22.93 (9.23)	3.65	0.057	0.011

*Significant at Bonferroni adjusted correction *p* =.0045 (in gender analysis)

Due to the small number of parents identifying as non-binary (*N* = 3) or transgender (*N* = 1), only male and female identifying parents were included in gender analysis. Results of a further one-way MANOVA analysis indicated a statistically significant difference in experiences between male and female parents, with a large effect size F(1, 342) = 5.47, *p* <.001, Pillai’s Trace = 0.141, η_p_^2^ = 0.141. Males reported more ITs, parenting stress, depression, and anxiety. Females were observed to have higher perceived parental competence (both self-efficacy and satisfaction) than males. No significant gender differences were observed for PLEs or stress. Table 7 details full results. A Chi-Square test shows more males were in the ‘potential risk’ PLE group χ(1) = 5.85, *p* =.016, the clinical range for depression χ(4) = 18.81, *p* = < 0.001, and anxiety χ(4) = 9.73, *p* =.045, but not stress χ(4) = 5.88, *p* =.209.

### Association with Parenting Experiences and Mental Health

A Pearsons correlation analysis was run to determine the relationship between ITs and PLEs, to perceived parental competence, parenting stress, and mental health symptoms (Table 8). As anticipated, strong correlations were seen between ITs and PLEs. Significant positive correlations were found between higher levels of ITs and PLEs, and higher scores on parenting stress, and mental health symptoms on the DASS-21. Significant negative correlations were found between ITs and PLEs to perceived parental competence. Correlation coefficients indicate small to medium effect sizes.

**Table 8:** Correlations of study variables

Variable	1	2	3	4	5	6	7	8	9	10	11
1. PTBC Thoughts											
2. PTBC Behaviours	0.815^**^										
3. PQ-16 Endorsement	0.489^**^	0.441^**^									
4. PQ-16 Distress	0.555^**^	0.511^**^	0.905^**^								
5. PSOC Total	− 0.432^**^	− 0.408^**^	− 0.382^**^	− 0.411^**^							
6. PSOC Satisfaction	− 0.357^**^	− 0.355^**^	− 0.401^**^	− 0.415^**^	0.833^**^						
7. PSOC Self-Efficacy	− 0.364^**^	− 0.326^**^	− 0.238^**^	− 0.272^**^	0.835^**^	0.391^**^					
8. PSS Total	0.336^**^	0.355^**^	0.299^**^	0.339^**^	− 0.811^**^	− 0.699^**^	− 0.655^**^				
9. DASS Depression	0.487^**^	0.472^**^	0.553^**^	0.619^**^	− 0.617^**^	− 0.595^**^	− 0.434^**^	0.603^**^			
10. DASS Anxiety	0.505^**^	0.476^**^	0.523^**^	0.581^**^	− 0.398^**^	− 0.391^**^	− 0.273^**^	0.359^**^	0.734^**^		
11. DASS Stress	0.538^**^	0.482^**^	0.537^**^	0.582^**^	− 0.540^**^	− 0.539^**^	− 0.363^**^	0.497^**^	0.792^**^	0.726^**^	

Note. ** Correlation is significant at *p* <.001 (2-tailed)

*PTBC* Parental Thoughts and Behaviours Checklist (thoughts and behaviour scales), *PQ-16* Prodromal Questionnaire-16-items (symptom endorsement and distress), *PSOC *Parental Sense of Competency Scale (Self-Efficacy and Satisfaction Subscales),*PSS * Parental Stress Scale,*DASS-21* Depression Anxiety Stress Scale

Multiple hierarchical regression analyses were conducted to explore if ITs (thoughts and behaviours) and PLEs (endorsement and distress) were associated with (1) perceived parental competence and (2) parenting stress, whilst controlling for mental health symptoms (anxiety, depression, stress) and other characteristics known to impact mental health: (a) birth trauma, (b) mental health history and (c) mental health treatment. A Bonferroni-adjusted alpha level of 0.007 was applied to the analysis of each predictor to correct for multiple analysis.

In the perceived parental competence model, ITs and PLEs were entered into step one (F(4, 344) = 26.54, *p* = < 0.001, R^2^ = 0.236), mental health symptoms into step two (F(7, 341) = 36.42, *p* = < 0.001, R^2^ = 0.428) and birth trauma, mental health history and mental health treatment into step three (F(10, 338) = 26.41, *p* = < 0.001, R^2^ = 0.439). All models significantly predicted parental competence. Variability increased by 2.03% in step three, suggesting these variables add to the prediction. Depression and anxiety, were found to be significant predictors of perceived parental competence in model 3, suggesting they drive the relationship between ITs, PLEs and parental competence.

In the parenting stress model, ITs and PLEs were entered into step one (F(4, 343) = 16.48, *p* = < 0.001, R^2^ = 0.161), mental health symptoms into step two (F(7, 341) = 32.24, *p* = < 0.001, R^2^ = 0.398) and birth trauma, mental health history and mental health treatment into step three (F(10, 338) = 23.77, *p* = < 0.001, R^2^ = 0.413). All models significantly predicted perceived parental competence. Variability increased by 2.5% in step three, suggesting these variables add to the prediction. Depression and anxiety were found to be significant predictors of parenting stress in model 3, suggesting they drive the relationship between ITs, PLEs and parenting stress. Table 9 details full results.

**Table 9:** Hierarchical regression analysis results

	F-Statistic	SE	*p*-value	*R* ^2^	*R*^2^Δ
Parental Competence Model 1 Model 2 Model 3	26.54 36.42 26.41	1.53 1.52 3.91	< 0.001 < 0.001 < 0.001	0.236 0.428 0.439	0.227 0.416 0.422
Parenting Stress Model 1 Model 2 Model 3	16.48 32.24 23.77	1.35 1.31 3.73	< 0.001 < 0.001 < 0.001	0.161 0.398 0.413	0.151 0.386 0.396
Outcome Variable	**Predictor Variable**	**B**	***β***	**t-value**	**p-value**
Parental Competence (Model 3)	(Constant)	88.98		22.74	< 0.001
	PTBC Thoughts	− 0.522	− 0.131	−1.74	0.083
	PTBC Behaviours	− 0.303	− 0.081	−1.13	0.258
	PQ-16 Endorsement	− 0.283	− 0.074	− 0.771	0.441
	PQ-16 Distress	0.126	0.067	0.629	0.530
	Depression	− 0.717	− 0.581	−7.77	< 0.001*
	Anxiety	0.262	0.186	2.74	0.006*
	Stress	− 0.189	− 0.142	−1.91	0.057
	Birth Trauma	−2.52	− 0.094	−2.21	0.028
	Mental Health History	− 0.646	− 0.024	− 0.523	0.602
	Mental Health Treatment	−1.20	− 0.039	− 0.802	0.423
Parenting Stress (Model 3)	(Constant)	26.27		7.79	< 0.001
	PTBC Thoughts	− 0.020	− 0.006	− 0.079	0.937
	PTBC Behaviours	0.464	0.148	2.02	0.045
	PQ-16 Endorsement	− 0.082	− 0.026	− 0.260	0.795
	PQ-16 Distress	− 0.048	− 0.030	− 0.276	0.573
	Depression	0.698	0.672	8.77	< 0.001*
	Anxiety	− 0.229	− 0.192	−2.77	0.006*
	Stress	0.142	0.126	1.66	0.097
	Birth Trauma	1.65	0.073	1.67	0.096
	Mental Health History	1.59	0.071	1.50	0.135
	Mental Health Treatment	1.55	0.060	1.20	0.230

Note.R^2^ΔR^2^adjusted;*SE* Standard Error,*B*Unstandardised Regression Coefficient,*β*Standardised Regression Coefficient,*PTBC*Parental Thoughts and Behaviours Checklist,*PQ-16*Prodromal Questionnaire-16-items

*Significant at Bonferroni adjusted correction of*p <*.007

**Fig. 1:**
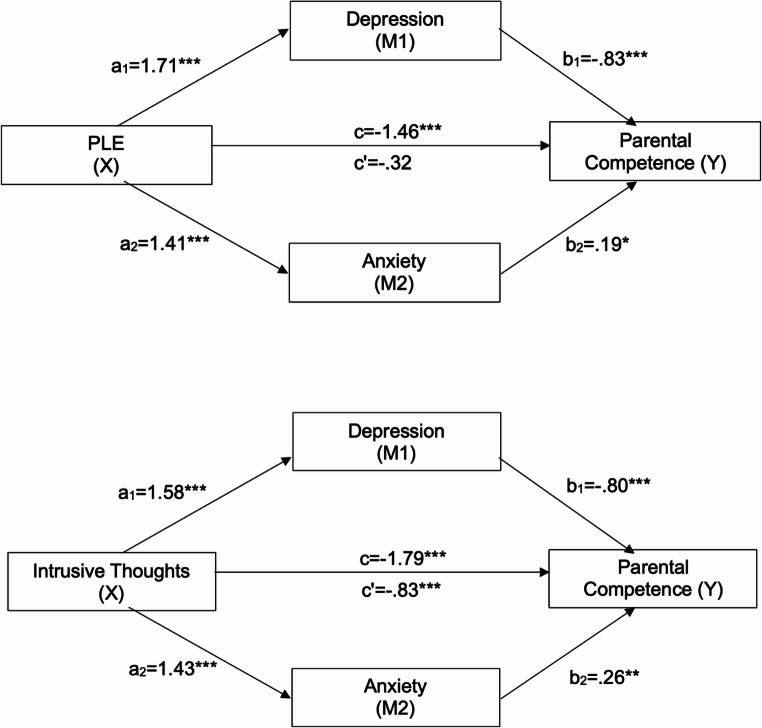
Multiple mediation analysis of the effect of PLEs and ITs on perceived parental competence, mediated by depression and anxiety. ***p<.001, **p<.01, *p<.05

**Fig. 2:**
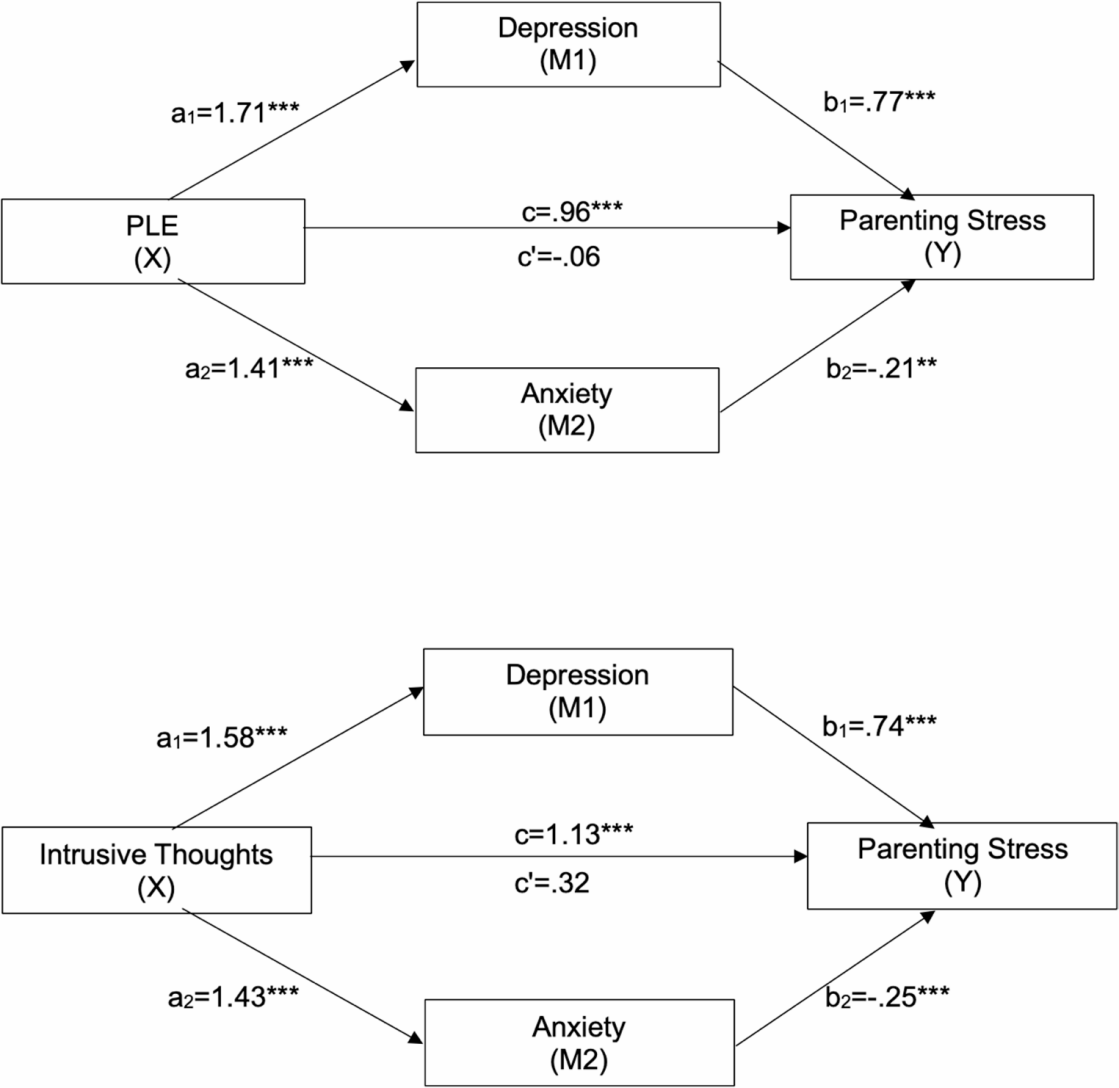
Multiple mediation analysis of the effect of PLEs and ITs on perceived parenting stress, mediated by depression and anxiety. ***p<.001, **p<.01

Mediation analyses were conducted to further explore the relationship between ITs and PLEs to parenting, whilst controlling for depression and anxiety. See Figs. 1 and 2. The direct effect of PLEs to perceived parental competence is not significant when considering anxiety and depression as mediators, however the direct effect of ITs to perceived parental competence remains significant when considering anxiety and depression as mediators. The direct effect of PLEs and ITs to parenting stress is not significant when considering anxiety and depression as mediators.

## Discussion

In a community sample of postnatal parents, ITs and PLEs were found to be prevalent, frequent and distressing. These experiences were associated with lower perceived parental competence, higher parental stress and increased symptoms of depression, anxiety, and stress. ITs and PLEs were also more frequently reported and distressing for parents who had experienced birth trauma and those with a history of mental health difficulties. Frequent and distressing ITs and PLEs were associated with higher parenting stress and lower perceived parental competence, although this relationship was indirectly mediated by depression and anxiety. Males experienced significantly more ITs, parenting stress, depression, anxiety, and lower perceived competence than females. Males were also more likely to fall into the ‘potential-risk’ for psychosis group, though given the small number of males recruited findings regarding gender should be interpreted with caution.

Most parents in our study reported current ITs and PLEs in the 12 months since birth, in line with existing findings (Abramowitz et al., 2006; Collardeau et al., 2019; Holt et al., 2018). We also found 30.4% participants to be considered at ‘potential-risk’ for psychosis. Those at ‘potential risk’ were more likely to experience more frequent PLEs and related distress, more frequent ITs, distress and related behaviours, lower perceived parental competence, increased parenting stress and more symptoms of depression, anxiety and stress than the ‘low risk’ group.

We found 88% of participants reported PLEs to be distressing, a far greater number than the 10% reported by Mannion and Slade (2014). Fairbrother and Woody (2008) reported minimal or low distress related to ITs, and Abramowitz et al. (2003) reported most parents experienced ITs to be mildly distressing; this is in stark contrast to the current study, where 90% indicated distress related to ITs. This difference may be due to a higher proportion of the current sample scoring in the clinical range for depression and anxiety than seen in previous perinatal research (Miller et al., 2006). In addition, almost half of the sample reported a history of mental health difficulties, and a quarter were awaiting or receiving treatment for current mental health difficulties. Nevertheless, when removing these participants, endorsement rates remained high: 81% endorsed at least one PLE and 83% endorsed at least one IT.

We found distressing ITs and PLEs were associated with increased parenting stress and lower perceived parental competence and that this relationship was indirectly influenced by depression and anxiety. This could suggest associations between ITs/PLEs and parenting outcomes, are largely explained by co-occurring internal symptoms, rather than reflecting unique predictors of parenting impairment. Given PLEs and ITs have been linked to greater anxiety and depression (MacKinnon et al., 2017; Mannion & Slade, 2014) and vice versa, it is unsurprising we found high correlations between these variables. Our findings support those of Thiséus et al. (2019) who found ITs were associated with anxiety, depression and increased parenting stress; and Fairbrother and Woody (2008) who found parenting stress was linked to ITs. In our sample, a large proportion of parents scoring within the clinical ranges for anxiety and depression also scored above the cut off for potential psychosis risk. This suggest that parents experiencing ITs and PLEs are more likely to also experience clinically significant symptoms of anxiety and depression. We found parents who felt more anxious and depressed, also feel less competent and satisfied in their parental role and experienced more parenting stress and vice versa. Complex mental health difficulties have been linked with the presence of PLEs (Stochl et al., 2015). Whilst PLEs may not indicate psychosis risk specifically, they may highlight more complex mental health presentations. It is important to consider self-perceived parenting competence is distinct from actual competence, and likely that parents experiencing mental health symptoms may be more likely to judge their own parenting harshly and doubt their competence.

Whilst we recognise perinatal OCD and psychosis are distinct disorders, our findings support those that suggest symptoms of OCD (such as ITs) and symptoms of psychosis (such as PLEs) can occur in the postnatal period (Rose et al., 2023). It could be useful to view findings though a transdiagnostic lens; growing evidence (Forbes et al., 2023) highlights symptom-level overlap could reflect shared underlying processes. It is important to avoid over-pathologizing subclinical experiences like ITs and PLES; whilst distressing, these experiences may function as markers of broader psychological vulnerability in the perinatal period and align with dimensional models of psychopathology (Forbes et al., 2023).

The current study identified possible differences in experiences between male and female parents. Albeit we recruited a small number of males, they reported higher levels of ITs, parenting stress, depression, anxiety, and lower perceived competence. This highlights the importance of considering the wellbeing of males in the perinatal period. Our findings contrast to those of Fairbrother et al. (2019) who found no gender differences in the number of ITs experienced. To our knowledge, no literature has explored PLEs in males during the postnatal period and our findings suggest further research is necessary.

### Strengths and Limitations

To our knowledge, this is the first study to investigate both ITs and PLEs in the postnatal period, with particular focus on frequency and distress as opposed to prevalence alone. We aimed to be inclusive of all parents and recruited a large sample (*N* = 349).

The study used self-report measures, and the sample was self-selecting, therefore response and selection bias may have occurred. Measurement of parenting experiences are likely a biased appraised when made by distressed parents. The online advertisement targeted parenting related social media which may have meant parents experiencing mental health concerns and distress were more inclined to complete the survey. This could mean our results include an over-reporting of experiences, which could impact the prevalence estimates and generalisability of our findings; ultimately, whilst important, findings should not be overstated, and further research will be helpful in consolidating prevalence estimates. It is unfortunate that a perinatal specific measure of psychosis was not available during the study, and we recognise limitations in using the PQ-16 as some items could be considered ‘normal’ perinatal experiences e.g., changes in smell could be related to postpartum rather than a PLE. However, we maintain that this tool is a useful screening tool for PLEs and highlighting those who could be at greater risk of developing later psychosis; it is not a diagnostic tool, and we did not use it as such.

Due to a small number of participants identifying as non-binary and transgender, gender comparison was made only between male and female parents.

We identified a percentage of participants who fell into the ‘potential risk’ group; whilst support resources were provided in the debrief form, due to the anonymity of the study, unfortunately specific support or intervention opportunities for this group were not directly offered.

Moreover, a cross-sectional design was applied where all variables were assessed simultaneously; causal relationships between study variables remain unclear. The presence of IT and PLEs alone does not necessarily result in adverse outcomes, however interpretation of these as distressing experiences can impact factors such as parenting and other wellbeing symptoms. It is likely the relationship between mental health and parenting experiences is reciprocal, with high stress and low perceived competence also exacerbating mental health difficulties. Longitudinal research is warranted to further explore the nature of the associations between these variables.

It would have been helpful to collect additional demographic information e.g., ethnicity, socioeconomic status, education level, and further detail regarding mental health history e.g. previous diagnosis/treatment; literature highlights how cultural context can shape how people interpret and cope with ITs and PLES (Larsen, 2004), without this information, it is challenging to understand how representative the sample is or how cultural factors may have influenced responses. Finally, information regarding previous pregnancy loss, and a specific infant age (postpartum stage) would have been useful to know, given these factors can impact PMH (Ahmed et al., 2019; Herbert et al., 2022; Vanwetswinkel et al., 2022).

### Implications

Existing research highlights up to 90% of the general population can experience ITs, indicating the presence alone is not necessarily the problem. Our findings suggest that for parents in the postnatal period, ITs and PLEs are not only prevalent, but frequent and interpreted as distressing. They can be linked to parenting experiences and could be more severe and distressing in parents with symptoms of depression, anxiety, and stress, and those who have experienced birth trauma and/or who have a history of mental health difficulties.

Healthcare professionals should routinely screen for a range of PMH symptoms, to facilitate early detection of parents experiencing distress and those at risk of potential adverse outcomes. Almost one-third of parents scored above the cut-off for potential psychosis risk and who also scored in the clinical range for depression and anxiety. This highlights how, even in community samples, clinical levels of symptoms are present. Professionals should be mindful that parents presenting with mental health difficulties and self-reported challenges with the parenting experience could also be experiencing ITs and PLEs. Increased training about ITs, PLEs and their associations with parenting and mental health, to professionals including GP’s, midwives, health visitors and healthcare assistants, will be important in identifying parents in need of additional support.

At a wider societal level, increased education regarding PMH, ITs and PLEs are important in normalising the prevalence of these experiences in the postnatal period and helping others to better understand the mechanisms behind related distress. Increased public awareness and conversation around ITs and PLEs is helpful in de-stigmatising the experiences and reducing treatment barriers (Clark et al., 2021). By showing that ITs and PLEs are common and frequent in the perinatal period, and not necessarily indicative of mental illness, parents may be more open to disclosing their experiences. Interventions including peer support groups (Jones et al., 2014), digital support tools (Baumel, 2023) and parenting marketing campaigns could also incorporate sharing of experiences and understanding. However, it is also important that we recognise that, for some, these experiences can transition into the clinical range and may require treatment.

Our findings highlight the importance of considering the mental wellbeing of male parents in the postnatal period, who may be less likely to engage with perinatal services or research (Philpott et al., 2019), may perceive services as not being accessible to them (Baldwin et al., 2019), or prioritise their partners’ wellbeing over their own (Darwin et al., 2017). More broadly, the associated stigma regarding male mental health can impact help-seeking behaviour (Bradbury, 2020). Therefore, all parents irrespective of gender identity, should be screened for PMH difficulties and mental health history should be considered.

Our results suggest subclinical symptoms of OCD (such as ITs), and also subclinical symptoms of postpartum psychosis (such as PLEs), occur in community perinatal populations, with varying levels of associated distress. This study explored subclinical symptoms only, therefore the full continuum of symptomology of these disorders within this population is not known but is an important area for future research. Literature suggests those experiencing persistent PLEs are at increased risk of developing psychiatric disorders (Dominguez et al., 2011), although transition rates to psychosis remain low in the general population (Werbeloff et al., 2012).

## Future Research

Future research can expand on the current study by applying a longitudinal design, to explore if and how experiences of distressing ITs and/or PLEs change over the perinatal period and what factors influence this. Future studies could benefit from adopting mixed-method designs to incorporate qualitative methodology to allow for richer information and understanding of these experiences. Wider advertisement studies of this nature (e.g., at baby groups) may produce a more representative sample. Additionally, studies should collect information about parents appraisals of ITs and PLEs and distress, given that cognitive theories of OCD and psychosis (Garety et al., 2001; Salkovskis, 1999) suggest appraisals are linked to distress; this can also aid understanding about transition rates from non-clinical to clinical level symptoms. Finally, researchers should aim to recruit larger samples of male, non-birthing parents and those who do not typically ‘fit’ the gender binary to better understand their experiences, which have historically been neglected in research.

## Conclusions

Postnatal ITs and PLEs can be distressing experiences for parents and are associated with lower perceived parental competence and satisfaction, increased parenting stress and other mental health symptoms. Depression and anxiety may indirectly influence the relationship between ITs, PLEs and parenting outcomes. Experiences may differ between female and male parents. Our findings suggest subclinical symptoms of perinatal OCD (including ITs) and subclinical symptoms of postpartum psychosis (including PLEs) are not only prevalent in community perinatal samples but are frequent and distressing. These findings highlight the importance of exploring and detecting a range of PMH symptoms in postnatal parents and supporting those who experience distress to prevent negative longer-term outcomes for parents and babies.

## Data Availability

The data that support the findings of this study are available from the corresponding author upon reasonable request.
